# Targeted Deletion of Centrin in *Leishmania braziliensis* Using CRISPR-Cas9-Based Editing

**DOI:** 10.3389/fcimb.2021.790418

**Published:** 2022-02-17

**Authors:** Rohit Sharma, Francys Avendaño Rangel, João Luís Reis-Cunha, Larissa Pinheiro Marques, Claudio P. Figueira, Pedro B. Borba, Sayonara M. Viana, Tom Beneke, Daniella C. Bartholomeu, Camila I. de Oliveira

**Affiliations:** ^1^Instituto Gonçalo Moniz, Fiocruz, Salvador, Brazil; ^2^Programa de Pós-graduação em Ciências da Saúde, Faculdade de Medicina da Bahia, Universidade Federal da Bahia, Salvador, Brazil; ^3^Departamento de Medicina Veterinária Preventiva, Escola de Veterinária, Universidade Federal de Minas Gerais, Belo Horizonte, Brazil; ^4^Departamento de Parasitologia, Federal University of Minas Gerais, Belo Horizonte, Brazil; ^5^Sir William Dunn School of Pathology, University of Oxford, Oxford, United Kingdom; ^6^INCT—Instituto de Investigação em Doenças Tropicais, Salvador, Brazil

**Keywords:** LeishGEedit, leishmaniasis, genetic manipulation, attenuation, vaccine development

## Abstract

*Leishmania braziliensis* is the main causative agent of Tegumentary Leishmaniasis in the Americas. However, difficulties related to genome manipulation, experimental infection, and parasite growth have so far limited studies with this species. CRISPR-Cas9-based technology has made genome editing more accessible, and here we have successfully employed the LeishGEdit approach to attenuate *L. braziliensis*. We generated a transgenic cell line expressing Cas9 and T7 RNA polymerase, which was employed for the targeted deletion of centrin, a calcium-binding cytoskeletal protein involved in the centrosome duplication in eukaryotes. Centrin-deficient *Leishmania* exhibit growth arrest at the amastigote stage. Whole-genome sequencing of centrin-deficient *L. braziliensis* (*LbCen^−/−^*) did not indicate the presence of off-target mutations. *In vitro*, the growth rates of *LbCen^−/−^* and wild-type promastigotes were similar, but axenic and intracellular *LbCen^−/−^* amastigotes showed a multinucleated phenotype with impaired survival following macrophage infection. Upon inoculation into BALB/c mice, *LbCen^−/−^* were detected at an early time point but failed to induce lesion formation, contrary to control animals, infected with wild-type *L. braziliensis*. A significantly lower parasite burden was also observed in mice inoculated with *LbCen^−/−^*, differently from control mice. Given that centrin-deficient Leishmania sp. have become candidates for vaccine development, we propose that *LbCen^−/−^* can be further explored for the purposes of immunoprophylaxis against American Tegumentary Leishmaniasis.

## Introduction

Human leishmaniasis results in mortality and morbidity worldwide, contributing to ~1 million new cases each year and placing 350 million individuals at risk of infection (Burza et al., 2018). Brazil alone reported 15,498 cases of Cutaneous/Mucosal Leishmaniasis in 2019 (https://www.paho.org/data/index.php/es/mnu-topics/leish-es/555-art-leish-es.html), the majority of which were caused by *Leishmania braziliensis*. Protection against *Leishmania* infection is dependent on the generation of IFN-γ-producing CD4^+^ T cells, leading to macrophage activation and parasite killing. Although infection with live parasites generates immunity, no vaccine against human leishmaniasis is yet available (Sundar and Singh, 2014). To this end, numerous attempts have been undertaken including but not limited to immunization with (i) live whole parasites, (ii) killed whole parasites, (iii) *Leishmania*-specific antigen and (iv) parasites attenuated by genetic modification [reviewed in (Zutshi et al., 2019)].

Following the first successful attempt of gene replacement in *L. major* (Cruz et al., 1993), genetic manipulation has now been achieved in different leishmania species. Since the recent introduction of CRISPR-Cas9-based methodologies, a significant advance has been made in this field: CRISPR-Cas-9 enabled the deletion of hundreds of genes, including, BTN1 (Ishemgulova et al., 2018) and LeishIF4E-3 (Shrivastava et al., 2019), genes encoding protein kinases (Baker et al., 2021) and flagellar proteins (Beneke et al., 2019) in *L. mexicana*, as well as LPG2 in *L. infantum* (Jesus-Santos et al., 2020), RAD51 in *L. major* (Damasceno et al., 2020) and Ros3 in *L. braziliensis* (Espada et al., 2021). Herein, we have used the LeishGEdit tool box (Beneke et al., 2017; Beneke and Gluenz, 2019) to manipulate the *L. braziliensis* genome. Parasites were initially engineered to express the Cas9 nuclease and the T7 RNA polymerase episomally. Thereafter, linear sgRNA and donor DNA constructs encoding resistance markers were transfected in parallel, allowing for single guide (sg) RNA transcription *in vivo* and, thus, integration of donor DNA cassettes within homology flanks, identical to the target locus. The advantages of the LeishGEdit approach are that cloning procedures, PCR purifications or *in vitro* transcription before transfection are unnecessary with the advantage that it can also be used for high throughput screening of mutants (Beneke et al., 2019; Damianou et al., 2020; Baker et al., 2021).

The centrin gene product is one of the several regulatory proteins required for duplication or segregation of the centrosome in higher eukaryotes and of basal bodies in lower eukaryotes (Wiech et al., 1996). Centrin is described as one of the essential factors in cell division in *Leishmania* (Selvapandiyan et al., 2001) and, thus, a centrin-deficient (centrin^−/−^) *L*. *donovani* mutant displays abnormal cytokinesis due to impaired centrosome function, leading to cell cycle arrest in the G2/M phase and the formation of multinucleated cells in axenic and intracellular amastigotes (Selvapandiyan et al., 2004). Importantly, immunization with centrin^−/−^ *L. donovani* provided strong protection against challenge with live parasites and immunized mice developed a multifunctional T cell response accompanied by a significant reduction in parasite load (Selvapandiyan et al., 2009). Recently, these findings have been expanded to show that immunization with centrin-deficient *L. major* confers protection against infected sand flies (Zhang et al., 2020b; Karmakar et al., 2021).

Based on the potential of centrin-defficient *Leishmania* to be developed as a vaccine leishmaniasis, we employed the LeishGEdit toolbox for targeted centrin deletion in *L. braziliensis*. Centrin deletion was precise without apparent off target effects and amastigotes from *L. braziliensis* lacking centrin amastigotes were multinucleated, akin to *L. donovani* centrin^−/−^ (Selvapandiyan et al., 2004). *L. braziliensis* amastigotes lacking centrin displayed impaired *in vitro* survival and, *in vivo*, they failed to induce lesion development *in vivo*. These results show that centrin-deficient *L. braziliensis* displays an attenuated behavior opening the possibility of immunoprophylaxis against New World Tegumentary Leishmaniasis.

## Materials and Methods

### Ethics Statement

Female BALB/c mice aged 6–8 weeks were obtained from the IGM/FIOCRUZ animal facility where they were maintained under pathogen-free conditions. All animal experimentation was conducted in accordance with the Guidelines for Animal Experimentation established by the Brazilian Council on Animal Experimentation (CONCEA). The local institutional review board (CEUA) approved all procedures involving animals (CEUA-015/2019-IGM/FIOCRUZ).

### Parasite Culture

*L. braziliensis* promastigotes (MHOM/BR/01/BA788) (De Moura et al., 2005) were maintained in Medium 199 (Sigma-Aldrich) supplemented with 20% heat-inactivated fetal calf serum (FCS), HEPES (40 mM), adenine (0.1 mM), Hemin (5 μg/ml), biotin (1 μg/ml) and antibiotics (penicillin 100 IU/ml and streptomycin 100 μg/ml) (all from Thermo Scientific) at 26°C. *L. braziliensis* transfectants expressing Cas9 and T7 RNA polymerase (*LbCas9T7*) or centrin^−/−^ mutants (*LbCen*^−^*^/^*^−^) were maintained in medium supplemented with hygromycin (100 µg/ml), or neomycin (50 µg/ml) and puromycin (10 µg/ml), respectively. Prior to *in vitro* and *in vivo* infection assays, parasites were incubated in Schneider`s insect medium (Sigma-Aldrich) supplemented with 10% FCS, 2 mM L-glutamine, penicillin (100 U/ml) and streptomycin (100 μl/ml) (Thermo Scientific). In *LbCas9T7*, *LbCen*^−^*^/^*^−^ and wild-type *L. braziliensis* (*LbWT*) promastigotes, *in vitro* parasite growth was determined by inoculating 5 × 10^5^ parasites/ml in supplemented Schneider’s medium at 26°C for 6 days. Parasite numbers were counted daily using a hemocytometer. *LbCas9T7*, *LbCen*^−^*^/^*^−^ and wild-type *L. braziliensis* (*LbWT*) axenic amastigotes were prepared transferring late promastigotes to a modified culture medium (20% FBS; pH 5.5) at 34°C, according to Teixeira et al. (2002). *In vitro* parasite growth was determined by inoculating 1 × 10^6^ parasites/ml in supplemented Schneider´s medium at 34°C for 5 days. Amastigote aggregates were disrupted by passing through a 25-gauge needle before counting in a hemocytometer.

### *In Silico* Identification of Putative *L. braziliensis* Centrin

The sequence of the putative *L. braziliensis* centrin gene (*LbrM.22.1290*) (*L. braziliensis* MHOM/BR/75/M2903) including flanking regions (3’ and 5’ FRs) was retrieved from TriTrypDB (http://tritrypdb.org) and this sequence was chosen because it presented the highest homology with previously characterized *L. donovani* centrin gene (GenBank—AF406767). The derived amino acid sequences from *L. donovani* and *L. braziliensis* centrin were aligned using the ClustalW program in the BioEdit software package (Version 7.0.4.1) and Geneious version 9.0 (https://www.geneious.com). The three-dimensional structure of putative centrin was predicted by the Phyre2 server using all default parameters. This server predicts 3D models using a single submitted protein sequence by gathering homolog sequences from a large non-redundant database, followed by secondary protein structure prediction [Hidden Markov Model (HMM)] (Kelley et al., 2015). The predicted 3D structure was visualized using the UCSF Chimera program (Pettersen et al., 2004). The quality of the predicted model was validated by Ramachandran Plot, generated using the PROCHECK server (Laskowski et al., 1996).

### Generation of *L. braziliensis* Overexpressing Cas9 and T7 (LbCas9T7)

Wild-type *L. braziliensis* promastigotes (*LbWT*) in mid-log phase (10^8^ cells/ml) were placed in Tb-BSF electroporation buffer (Schumann Burkard et al., 2011) and mixed with plasmid pTB007 (Beneke et al., 2017) (~5 µg) carrying the humanized *Streptococcus pyogenes* Cas9 nuclease gene (hSPCas9) and T7 RNA Polymerase (T7 RNAP) in a pre-chilled 2 mm electroporation cuvette (SIGMA). Electroporation was performed using a Bio-Rad Gene Pulser Xcell System followed by subsequent clonal selection in M199 medium supplemented with hygromycin (50 μg/ml) in 96-well cell culture plates. Cultures were monitored for 2–4 weeks and emerging transfectants were further expanded in selective medium at 100 μg/ml hygromycin.

To confirm Cas9 expression, cell lysates were prepared with 1 × 10^7^ mid-log parasites using NuPAGE sample reducing agent and LDS Buffer (Invitrogen) following manufacturer’s protocol. *LbWT* and *LbCas9T7* cell lysates were separated on NuPAGE mini protein gel (4–12%) (Invitrogen) and subsequently transferred onto a nitrocellulose membrane using the iBLOT system (Invitrogen). Membranes were blocked in 10% non-fat milk in PBS-T (Tween 20—0.1%) at 4°C, overnight. Following PBS-T washes, membranes were probed with mouse anti-Cas9 antibody (Clone 7A9, BioLegend); washed and probed with goat anti-mouse IgG-HRP (Clone Poly4053, BioLegend). Bound conjugates were detected by ECL western blotting reagents (Thermo Scientific) visualized under ImageQuant LAS 4000 (GE Healthcare).

### Generation of Centrin-Deficient *L. braziliensis* (LbCen^−/−^)

Gene deletion was performed as previously described (Beneke et al., 2017; Beneke and Gluenz, 2019). Briefly, commercially synthesized oligonucleotides were used to generate the DNA templates for target-specific single guide (sg) RNA (3`and 5`) containing the T7 promoter, the 20nt sgRNA target sequence and a sequence complementary to the sgRNA scaffold. Primers for the amplification of donor DNA containing the neomycin phosphotransferase II (NEO) and puromycin N-acetyltransferase (PAC) genes with target-specific 30nt homology flanks and G00 primer (sgRNA scaffold) were designed using LeishGEdit (www.leishgedit.net) (Beneke et al., 2017) (**Supplemental Table 1**) and commercially synthesized. To amplify the sgRNA template DNA, a mixture containing G00 primer (100 μM), high fidelity polymerase (Invitrogen), dNTPs and MgCl_2_ was prepared, then added to the diluted sgRNA primers (4 µM) and placed in separate tubes containing 5’ sg RNA and 3’ sg RNA primers (pre-frozen at −80°C for 10 min). PCR was performed on a preheated block under the following cycling conditions: 98°C for 30 s (1 cycle), 98°C for 10 s, 60°C for 30 s, 72°C for 15 s (35 cycle), 72°C for 10 min (1 cycle).

PAC and NEO donor DNA were amplified using the pTPuro or Neo plasmid templates, respectively (Beneke et al., 2017). Briefly, 0.5 μl (30 ng/μl stock) of each template was mixed with 8 μl of diluted forward and reverse primers (10 μM) and frozen at −80°C for at least 10 min. A master mix containing high-fidelity polymerase (Invitrogen), DMSO, dNTPs, MgCl_2,_ and nuclease-free water was prepared and added to the primer template mix. PCR was performed on a preheated block under the following cycle conditions: 98°C for 5 min (1 cycle), 98°C for 30 s, 65°C for 30 s, 72°C for 2 min (40 cycles), 72°C for 7 min (1 cycle). The amplification products of sgRNA templates and donor DNA were further confirmed by running samples on 2 and 1% agarose gel, respectively.

To generate *LbCen^−/−^*, transfection of Cas9 and T7 RNAP-expressing *L. braziliensis* (*LbCas9T7*) was performed as previously described (Beneke and Gluenz, 2019), with minor modifications. A mixture containing sgRNA templates and donor DNA was heated at 94°C for 5 min prior to transfection, mixed with 150 μl mid-log Cas9 T7-expressing *L. braziliensis* resuspended in transfection buffer (Tb-BSF; 10^8^ cells/ml) and transferred to a prechilled 2 mm cuvette. Transfection was performed using two pulses and the X-001 program on a Nucleofector™ 2b Device (Lonza Biosciences, USA). Transfectants were transferred to 5 ml M199 medium in a 25 cm^2^ flask and incubated for 24 h at 26°C. *LbCen^−/−^* clones were selected from plates containing M199 supplemented with 50 μg/ml of neomycin (PROMEGA) and 10 μg/ml of puromycin (SIGMA).

Following transfection and clonal selection, total genomic DNA from *LbWT, LbCas9T7* and *LbCen^−/−^* was isolated using PureLink Genomic DNA Mini Kit, according to manufacturer’s instructions (Invitrogen). *Centrin* deletion and integration of antibiotic resistance markers was confirmed by PCR using specific primers, designed using Geneious software (Biomatters) (**Supplemental Table 1**). PCR conditions employed were: (i) Centrin ORF detection: 94°C for 3 min (1 cycle), 94°C for 30 s, 53°C for 30 s, 72°C for 1 min (40 cycle), 72°C for 7 min (1 cycle). (ii) Neomycin and (iii) puromycin resistance cassettes: 94°C for 3 min (1 cycle), 94°C for 30 s, 55°C for 30 s, 72°C for 1 min (40 cycles), 72°C for 7 min (1 cycle). Amplicons were separated on 2% agarose gel and visualized using ImageQuant LAS 4000 (GE Healthcare).

### Genome Wide Analysis of *L. braziliensis* Overexpressing Cas9 and T7 (LbCas9T7) and of Centrin-Deficient *L. braziliensis* (LbCen^−/−^)

To confirm Cas9-mediated centrin deletion, whole genome sequencing (WGS) was performed in the *LbCas9T7* and *LbCen^−/−^* clones using an Illumina Hiseq2000 sequencer, with ~60× coverage, paired-end read libraries with a 100 bp read size and 350 bp insert size. FastQC (Andrews, 2010) and Trimmomatic (Bolger et al., 2014) were used for read quality control and adapter sequence removal, respectively. BWA-mem (Li and Durbin, 2009) was used to map the processed reads to the *L. braziliensis* MHOM/BR/75/M2904 v45 reference genome, obtained from TriTrypDB (Aslett et al., 2010). Reads with a mapping quality score <30 were discarded using SAMtools (Li et al., 2009). Confirmation of centrin deletion was performed based on read depth estimations and visualized *via* the Integrative Genomics Viewer (IGV) tool (Robinson et al., 2011) using bam files containing the aligned reads and the *L. braziliensis* MHOMBR75M2904 v45 General Feature Format (GFF), available at TriTrypDB (Aslett et al., 2010). The raw Illumina sequencing reads were deposited under the Bioproject accession number PRJNA763382.

To further confirm LbrM.22.1290 deletion and estimate the potential for off-target genomic alterations, the read depth of each *L. braziliensis* gene was estimated and normalized in the *LbCas9T7* and *Lb*Cen^−/−^ read libraries by the genome coverage using BedTools (Quinlan and Hall, 2010), and in-house Perl scripts. The absolute *LbCas9T7* and *Lb*Cen^−/−^ read depth difference of each gene was estimated using the R program (https://www.R-project.org/) and visualized using ggplot2 (Wickham, 2016). All genes with an absolute difference higher than 0.5 genome coverage, which corresponds to approximately one copy per haploid genome, were reported.

### Microscopy

Axenic *LbWT*, *LbCas9T7* and *LbCen*^−^*^/^*^−^ amastigotes were generated as described elsewhere (Teixeira et al., 2002). After 96 h in culture, axenic amastigote aggregates were disrupted and the cells then washed in PBS, fixed in 4% paraformaldehyde, washed again in PBS and resuspended in DAPI solution (1 µg/ml, Molecular Probes) for 30 min. Parasites were washed twice in PBS and resuspended in 100 µl of PBS. Parasites were centrifuged, stained and visualized under a TCS SP8 scanning laser confocal system (Leica). Alternatively, axenic amastigotes (48 h of culture) were fixed in Karnovsky fixative (2% glutaraldehyde and 2% paraformaldehyde in 0.1 M sodium cacodylate buffer, pH 7.4), washed in cacodylate buffer and then post-fixed with 1% osmium tetroxide (OsO_4_) in 0.1 M sodium cacodylate buffer. Samples were mounted on poly-Lysine-coated coverslips, and then dehydrated in an ascending ethanol series (30, 50, 70, 90, and 100%). Finally, samples were critical-point dried in CO_2,_ mounted on stubs, coated with gold (20–30 nm) and visualized by scanning electron microscopy (SEM). Axenic amastigotes were also evaluated by Transmission Electron Microscopy (TEM). After 48 h, axenic amastigotes (*LbWT*, *LbCas9T7*, and *LbCen^−/−^*) were washed in PBS, fixed and then post-fixed as described above. Samples were dehydrated in an acetone series (30, 50, 70, 90, and 100%) and embedded in Poly/Bed^®^ 812 resin. Ultrathin sections were obtained and mounted on 300-mesh grids, then stained in 5% uranyl acetate and lead citrate. Cells were analyzed at Jeol JEM 1230 transmission electron microscope and Jeol JSM 6390LV scanning electron microscope.

### *In Vitro* Infection

Primary macrophages obtained from mouse bone marrow were suspended in RPMI 1640 medium (Sigma-Aldrich), supplemented with 10% FBS, 2.5% HEPES, 100 IU/ml penicillin and 100 µg/ml streptomycin (all from Invitrogen) and seeded at 3 × 10^5^ cells/500 μl/coverslip per well on 24-well plates. Monolayers of adherent macrophages formed on the coverslips were washed to remove any non-adherent cells and then exposed to stationary phase *LbWT* and *LbCen^−/−^* at a 10:1 parasite/host cell ratio (clone #4), i.e., 3 × 10^6^/500 μl in RPMI 1640 + 10% FBS/well. The plates were incubated at 35°C under 5% CO_2_ for 24 h, and then coverslips were extensively washed to remove any non-internalized *Leishmania*. At different time periods after parasite exposure, coverslips were methanol-fixed and stained with hematoxylin and eosin (H&E). *Leishmania* loading or macrophage infection was assessed by scanning 200 macrophages in each sample for the enumeration of cells with and without *Leishmania*; the total number of intracellular parasites was counted by optical microscopy.

### *In Vivo* Infection

BALB/c mice (n = 10) were inoculated with 2 × 10^5^ *LbWT or LbCen^−/−^*. Parasites were inoculated into the left ear dermis in 10 μl PBS using a 27G needle. Parasite loads were determined at different time points by limiting dilution analysis in samples obtained from mouse ears, draining lymph nodes and spleen as previously described (Novais et al., 2013). Lesion development was monitored weekly by measuring the thickness of the ear using a digital caliper (Thermo).

### Statistical Analysis

Comparisons between two groups were performed using Mann–Whitney (non-parametric t-test), and among three of more groups using the Kruskal–Wallis test. Analyses were conducted using Prism software (V.8.0, GraphPad) and *p-*values ≤0.05 were considered significant. Data are presented as mean± standard deviation.

## Results

### *In Silico* Characterization of *L. braziliensis* Putative Centrin Gene (LbrM.22.1290)

A BLASTP search in the TriTrypDB database revealed the presence of a putative centrin gene in *L. braziliensis* (*LbrM.22.1290*) showing homology to previously characterized *L. donovani* centrin [GenBank—AF406767; (Selvapandiyan et al., 2001)]. The putative *L. braziliensis* centrin is 450 nucleotides (nt) long (including stop codon; *orf* −447 bp) and encodes a protein containing 149 amino acids (AAs) (**Supplemental Figure 1A**). Pairwise amino acid alignment between *LbrM.22.1290* and *L. donovani* centrin indicated high identity (94.63%) and high degree of conservation among the calcium binding sites (EF-hand 1 and 4; alpha helical stretch) (**Supplemental Figure 1B**).

**Figure 1:**
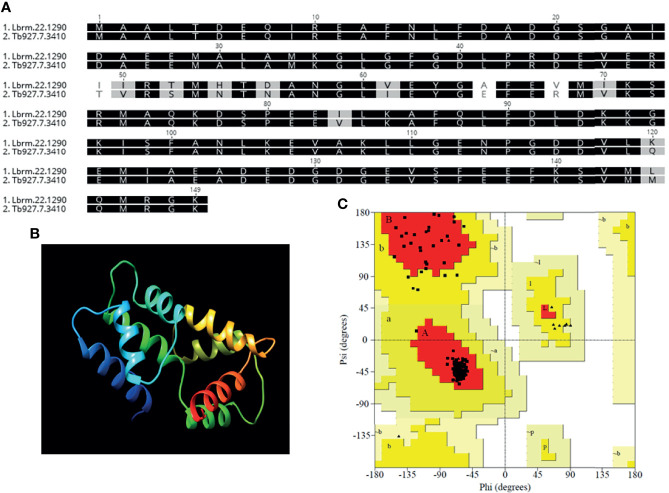
Homology modeling of the derived protein sequence from putative *L. braziliensis* centrin (LbrM.22.1290). **(A)** Amino-acid sequence alignment of *L. braziliensis* (Lbr.M.22.190) and *Trypanosoma brucei* (Tb927.7.3410) centrin used as the template for the structure prediction with 99.9% confidence and 100% sequence coverage. **(B)** Three-dimensional structure of the predicted protein and **(C)** Ramachandran analysis of the predicted *L. braziliensis* centrin: Red: most favored regions, Yellow: additional allowed, generously allowed regions and Light Yellow- disallowed regions.

The amino acid sequence of *LbrM.22.1290* was used to predict the *in silico* three-dimensional structure using the solved structure of *Trypanosoma brucei* centrin 4 (*Tb927.7.3410*). Again, the amino acid sequences in *LbrM.22.1290* and *Tb927.7.3410* showed high sequence homology (**Figure 1A**). Molecular modeling using 100% of *LbrM.22.1290* (149 AAs) allowed prediction of the protein structure with 99.9% confidence (**Figure 1B**). The stereochemical quality of the modeled structure was validated by Ramachandran plot analysis, revealing that 93.1 and 6.9% of the residues were in the most favored and additionally allowed regions, with no residues in disallowed regions (**Figure 1C**). These *in silico* analyses suggest that the putative centrin gene in *L. braziliensis* is a structural homolog of *T. brucei* centrin 4 and centrin 4 is highly conserved across *Leishmania* and *Trypanosoma* spp.

### CRISPR Cas9-Mediated Deletion of Centrin in *L. braziliensis*

**Figure 2:**
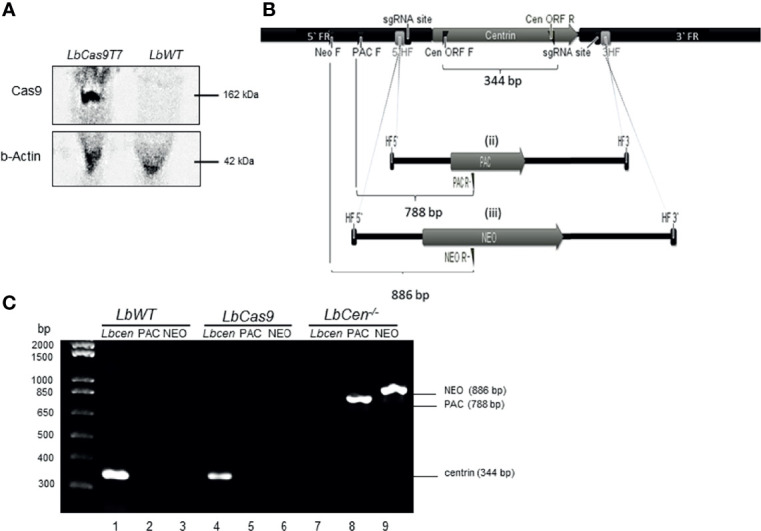
Generation of *centrin^−/−^ L. braziliensis*. **(A)** Western blot of whole cell lysates probed with anti-Cas9 antibody and anti-b-Actin. *LbWT*, parental cell line, *LbCas9T7*, *L. braziliensis* expressing Cas9 and T7. **(B)** *In silico* representation of the CRISPR-Cas9 based deletion of the *L. braziliensis* putative centrin gene (i) centrin genomic locus indicating sgRNA (guide RNA) binding sites at both 5` and 3` UTRs, 3`& 5` HF (homology flank) or flanking regions (30 bp) and primers for the correct integration (flag). PAC and NEO forward primers (PAC F and NEO F), Cen ORF forward and reverse (CEN ORF F and CEN ORF R) diagnostic primers for detection of centrin gene (amplification of 344 bp fragment). (ii) & (iii) Donor cassettes containing PAC and NEO antibiotics markers, indicating diagnostic reverse primers (PAC R and NEO R and size of expected amplicons for the confirmation of correct integration of the cassettes (788 and 886 bp, respectively). **(C)** PCR analysis of generated cell lines: test for the presence of the *Centrin* in Lb WT parental line and in LbCas9T7; test for the integration of the PAC and NEO-resistance genes in *LbCen^−/−^*. PCR products were analyzed on a 1% agarose gel.

Our strategy consisted in first generating an *L. braziliensis* line to co-express Cas9 and T7 RNAP (*LbCas9T7*). Following transfection of mid-log *LbWT* promastigotes with pTB007, carrying genes for Cas9 and T7 RNAP, transfectants were selected under antibiotic pressure (HYG) and resulting clones were grown in selective medium. Western blot analysis confirmed successful expression of the Cas9 protein (162 kDa) (**Figure 2A**). One resulting clone (*LbCas9T7*) was selected for deletion of centrin ORF. Next, *L. braziliensis* centrin alleles were targeted with PAC and NEO donor DNA cassettes, each flanked by 30 nt homologous sequences. Primers were designed for the identification of centrin^−/−^ *L. braziliensis* (*Lb*Cen^−/−^) line by PCR (**Figure 2B** and **Supplemental Table 1**). *LbCas9T7* parasites were first transfected with the two donor DNA constructs (PAC and NEO) plus two sgRNAs (DNA) templates targeting the 5’ and 3’ FRs of the centrin ORF. Following transfection and serial limiting dilution, clonal selection under antibiotic pressure (puromycin and neomycin) yielded six clones, which were further expanded. Centrin deletion was confirmed by PCR as shown by the absence of the centrin amplicon (344 bp *orf* fragment) paralleled by presence of amplicons specific for PAC (788 bp) and NEO (886 bp), indicating the successful integration of both donor cassettes (**Figure 2C**). Additionally, PCR products spanning the integration sites were Sanger-sequenced, confirming integration of both donor cassettes (**Supplemental Figure 2**). These data confirm the successful use of the LeishGEdit for deletion of the putative centrin gene (*LbrM.22.1290*) in *L. braziliensis*.

### Genome Wide Analysis of Centrin^−/−^ and Cas9-T7-Overexpressing *L. braziliensis*

**Figure 3:**
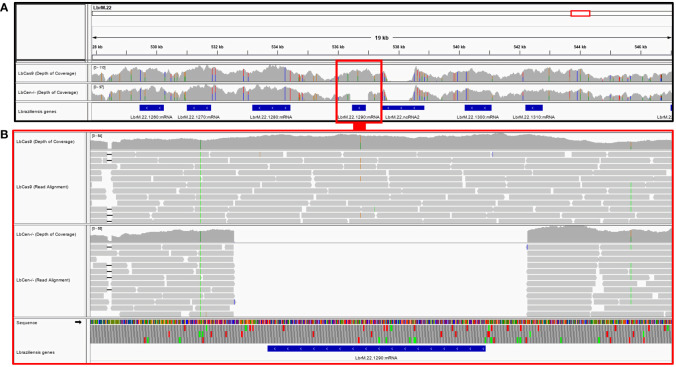
Deletion of the LbrM.22.1290 centrin gene as confirmed by whole genome sequencing. **(A)** MHOM/BR/75/M2904 *L. braziliensis* chromosome 22 encompassing the 527,855–547,147 region covered by *LbCas9T7* and *LbCen^−/−^* genomic read libraries. Blue boxes represent *L. braziliensis* genes, drawn in scale; LbrM.22.1290 centrin gene region is highlighted by red box. Gray histograms above the genes represent the read depth in each genomic position for each genomic library, where colored markings denote SNPs in the reads when compared to the reference genome. **(B)** Read mapping in the genomic region encompassing the LbrM.22.1290 gene. Mapping of individual reads is represented by gray boxes. An expected number of reads mapped into the LbrM.22.1290 centrin gene region in the *LbCas9T7* isolate, whereas no read from *LbCen^−/−^* library mapped into this gene.

**Figure 4:**
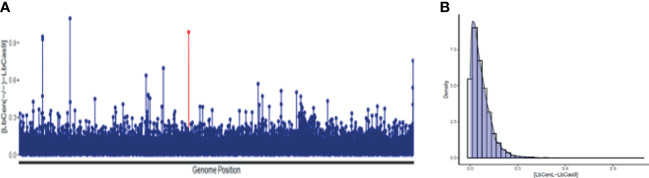
Read depth coverage (RDC) alterations in *LbCas9T7* and *LbCen^−/−^*. **(A)** Absolute values of the RDC difference from *LbCas9T7* and *LbCen^−/−^* genomic libraries in each *L. braziliensis* gene normalized by genome coverage. Each line of the X axis corresponds to a gene, ordered by its genome position from the smallest to the largest chromosome. The Y axis represents the difference of RDC absolute values for each gene from *LbCas9T7* and *LbCen^−/−^* lines. The LbrM.22.1290 centrin gene is highlighted in red. **(B)** Density plot of the RDC differences in *LbCas9T7* and *LbCen^−/−^* lines. The X axis corresponds to the difference of RDC absolute values of each gene from *LbCas9T7* and *LbCen^−/−^* lines, the Y axis represents the distribution of occurrence of these values. Most of the differences are below 0.3.

Next, we performed whole genome sequencing of *LbCas9T7* and *LbCen^−/−^* to verify the targeted deletion of *L. braziliensis* centrin in *LbCen^−/−^*. Initially, the read depth across the genome of *LbrM.22.1290* in both *LbCas9T7* and *LbCen^−/−^* was estimated and the only relevant alteration was the expected absence of coverage in the centrin gene region in *LbCen^−/−^* (**Figure 3**). These data show targeted centrin deletion and do not indicate translocation to another genomic locus. Also, allele frequency analysis of heterozygous SNPs revealed that, different from the *L. braziliensis* M2904 reference genome which essentially triploid (Rogers et al., 2011), the *L. braziliensis* BA788 genome is overall diploid, with chromosome 22 that harbors centrin gene having two copies (data not shown). Therefore, the successful generation of centrin knockout was achieved with two resistance markers. To evaluate the potential occurrence of Cas9-induced deletions or duplications in other genomic regions that could have an impact on phenotype, the read depth of all *L. braziliensis* MHOMBR75M2904 genes normalized by the genome coverage was estimated and compared, for both *LbCas9T7* and *LbCen^−/−^* (**Figure 4**). Most differences in read depth ranged from “0” to “0.3”, suggesting minimal fluctuations in the read libraries that are unlikely to result in a mutant-specific phenotype (**Figures 4A, B**). Read depth differences larger than 0.5 were only observed in 12 genes, all of which were multicopy genes. These 12 occurrences corresponded to five multicopy genes with a basal coverage higher than “3”, such as amastins, zinc-transporters and metallopeptidases. In addition, five were multi-copy structural RNAs, e.g., snoRNA and snRNA. One was a 40S ribosomal protein (LbrM.30.0750), and the other the expected centrin gene, *LbrM.22.1290* (**Table 1**). Taken together, these results do not indicate the presence of off-target deletions or duplications in the genome of the newly generated *LbCen^−/−^*.

**Table 1:** RDC differences between LbCas9T7 - LbCen−/−. For a total of 12 genes, LbCas9–LbCen^−/−^ RDC difference is higher than 0.5. From these, eleven correspond to multigene families or structural RNAs, and only one, the centrin LbrM.22.1290 gene, has the expected read depth coverage of a single copy gene in LbCas9 line, with RDC value of zero for LbCen^−/−^ line.

Genes	LbCas9T7	LbCen^−/−^	RDC [LbCen^−/−^ -LbCas9T7]	Annotation
LbrM.05.snoRNA2	5.3	6.2	0.9	LM5Cs1H1
LbrM.05.snRNA1	5.3	6.2	0.9	spliced_leader_associated_RNA%2C_SLA_RNA
LbrM.10.0480	7.8	6.7	1.0	metallo-peptidase%2C_Clan_MA(M)%2C_Family_M8
LbrM.20.0790	6.4	5.8	0.6	amastin-like_surface_protein%2C_putative
LbrM.20.4290	3.7	4.4	0.7	amastin-like_surface_protein%2C_putative
LbrM.22.1290	0.9	0	0.9	centrin-4%2C_putative
LbrM.28.2110	6.2	5.7	0.6	Zinc_transporter_3%2C_putative
LbrM.30.0750	1.0	1.6	0.5	40S_ribosomal_protein_S30%2C_putative
LbrM.31.0160	3.5	4.0	0.5	paraflagellar_rod_protein_1D
LbrM.35.snoRNA2	5.2	5.8	0.5	LM36Cs2H1
LbrM.35.snoRNA5	4.8	5.4	0.5	LM36C2C2
LbrM.35.snoRNA6	5.6	4.9	0.8	LM36C1C1

For a total of 12 genes, difference is higher than 0.5. From these, eleven correspond to multigene families or structural RNAs, and only one, the centrin LbrM.22.1290 gene, has the expected read depth coverage of a single copy gene in LbCas9T7 parasite, and RDC value of zero for LbCen−/− parasite.

### Phenotypic Characterization of Centrin-Deficient *L. braziliensis*

**Figure 5:**
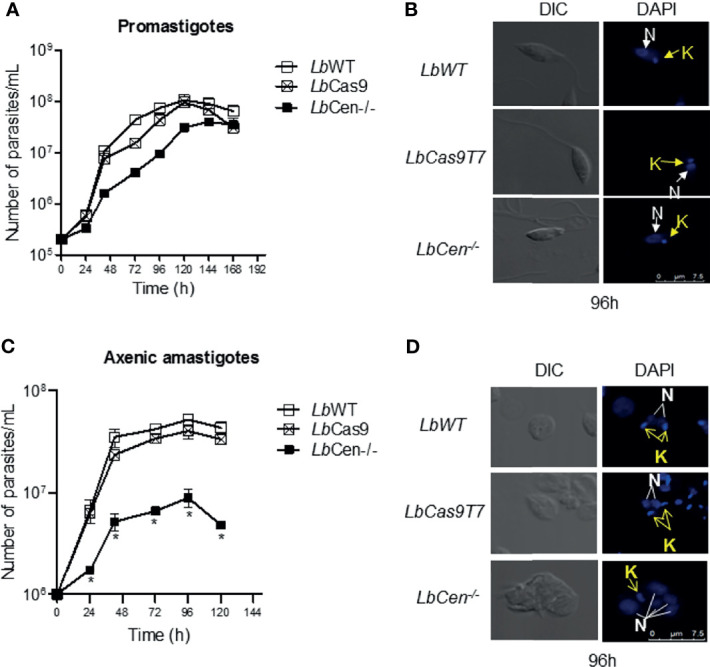
Kinetics of *LbCas9T7* and *LbCen^−/−^* promastigote growth *in vitro*. **(A)** Promastigote cultures were started at 5 × 10^5^ parasites/ml and were maintained at 26 °C for 7 days in supplemented Schneider media. Parasite numbers were determined daily by counting using a hemocytometer. Data is plotted as mean ± SEM and is from a representative experiment, performed in triplicate. **(B)** DIC and fluorescent (DAPI) representative micrographs of *Lb*WT, *LbCas9T7*, and *LbCen^−/−^* promastigotes after 96 h of culture. **(C)** Axenic amastigote cultures were started at 1 × 10^6^ parasites/ml and were maintained at 34°C for 5 days in supplemented Schneider media, pH 5.5. Parasite numbers were determined daily by counting using a hemocytometer. Data is plotted as mean ± SEM, and is from a representative experiment, performed in quadruplicate, *p < 0.05. **(D)** DIC and fluorescent (DAPI) representative micrographs of *Lb*WT, *LbCas9T7*, and *LbCen^−/−^* amastigotes after 96 h of culture.

Next, we evaluated the phenotype of *LbWT*, *LbCas9T7*, and *LbCen^−/−^*. Comparative promastigote growth curves showed similar proliferation rates over a 7-day time course (**Figure 5A**). Promastigotes (96 h cultures) were stained with DAPI and examined by confocal microscopy. *LbWT*, *LbCas9T7*, and *LbCen^−/−^* promastigotes did not exhibit any morphological changes differences and all three lines presented mostly single nuclei (**Figure 5B**). *LbCen^−/−^* axenic amastigotes, however, exhibited a significantly deficient growth, starting at 24 h and persisting over a 5-day time course, when compared to *LbWT* and *LbCas9T7* axenic amastigotes (**Figure 5C**). As expected, *LbCen^−/−^* amastigotes had multiple nuclei and kinetoplasts (**Figure 5D**). CRISPR-mediated deletion of centrin in *L. braziliensis* yielded a cell line that displayed slower growth at the amastigote stage, with similar morphological changes as those described in *L. donovani* centrin*^−^*^/^*^−^* mutants (Selvapandiyan et al., 2004).

**Figure 6:**
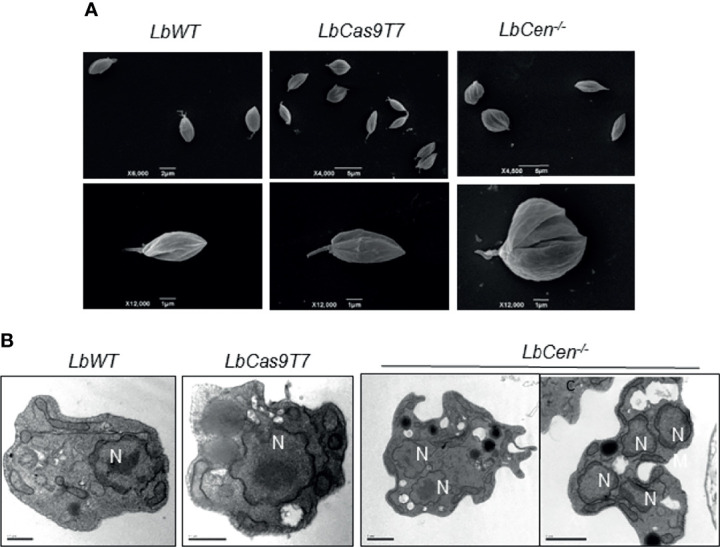
Ultrastructural analysis of *LbCen^−/−^* axenic amastigotes. *Lb*WT, *LbCas9T7*, and *LbCen^−/−^* axenic amastigotes were harvested, fixed and prepared for scanning **(A)** or transmission electron microscopy **(B)**. Transmission electron micrographs of axenic amastigotes showing the presence of a single nucleus (N) in *Lb*WT and in *LbCas9T7* and the presence of multi nuclei in *LbCen^−/−^*. Scale bars, 0.5 µm (*LbWT* and *LbCas9T7*); 1 µm (*LbCen^−/−^*).

*LbCen^−/−^* axenic amastigotes (48 h culture) were subjected to morphological analyses by SEM and TEM. SEM results showed the presence of abnormally large cells compared to the normal morphology observed in *LbWT* and *LbCas9T7* (**Figure 6A**). *LbCen^−/−^* axenic amastigotes (48 h culture) observed under TEM showed prominently large cells with multiple nuclei and/or kinetoplasts, suggesting a defective cytokinesis whereas both *LbWT* and *LbCas9T7* axenic amastigotes did not (**Figure 6B**). Thus, *LbCen^−/−^* exhibited similar morphology to that described for centrin*^−^*^/^*^−^ L. donovani* (Selvapandiyan et al., 2001; Selvapandiyan et al., 2004).

### *In Vitro* and *In Vivo* Infectivity of Centrin-Deficient *L. braziliensis*

**Figure 7:**
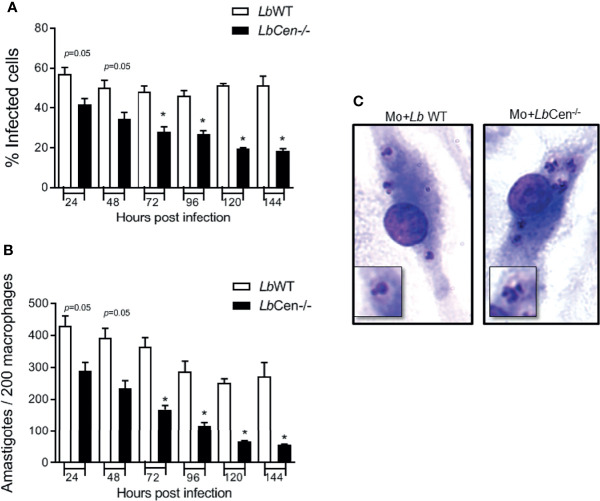
Macrophage infection with *LbCen^−/−^*. BMDM were infected with Lb*WT or LbCen^−/−^* (10:1, parasite/macrophage ratio) promastigotes for 24 h. Cells were extensively washed and further cultured for 48, 72, 96, 120 or 144 h. Cells were stained with H&E and evaluated for the percentage of infection **(A)** and the number of amastigotes per 200 macrophages **(B)** by optical microscopy. **(C)** Photomicrographs showing infected macrophages at 96 h. Data (mean ± SEM) are pooled from four independent experiments, each performed in quadruplicate. *p < 0.05.

Given the growth arrest (**Figure 5C**) and the prominent structural alterations (**Figure 6**) observed in axenic amastigotes, we predicted that *LbCen^−/−^* would also exhibit reduced infectivity in the host cell. A time course analysis of macrophage infection revealed a significant reduction in the percentage of cells infected with *LbCen^−/−^* compared to cells infected with *LbWT*, as early as 72 h after parasite exposure (**Figure 7A**). This result was paralleled by a significantly reduced number of amastigotes detected within macrophages at the same time point (**Figure 7B**). H&E staining of infected cells also confirmed the presence of multiple nuclei in cells infected with *LbCen^−/−^* compared to *LbWT* (**Figure 7C**). Therefore, CRISPR-Cas9-mediated deletion of centrin impairs the *in vitro* growth of *L. braziliensis*.

**Figure 8:**
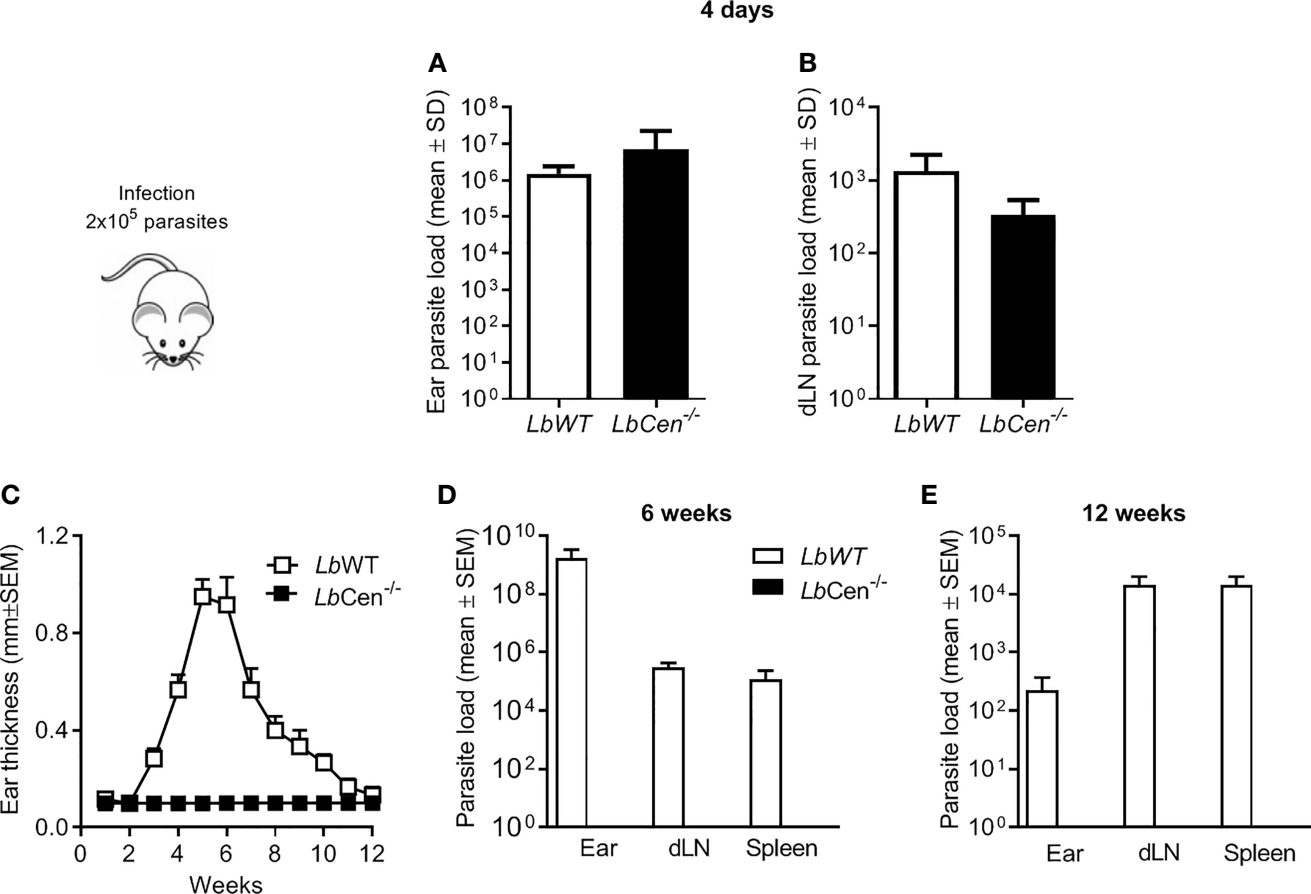
Parasite load in mice inoculated with *LbCen^−/−^*. BALB/c mice (10 per group) were infected with 2 × 10^5^ *LbWT* or *LbCen^−/−^* promastigotes, in the ear dermis and parasite load was determined, four days later, at the inoculation site (ear) **(A)** and in draining lymph nodes **(B)** by Limiting Dilution Analysis. Data (mean ± SEM) are from one representative experiment. BALB/c mice (10 per group) were infected as described and lesion development was measured weekly **(C)**. Six **(D)** and twelve **(E)** weeks post infection, parasite load was evaluated by Limiting Dilution Analysis. Data (mean ± SEM) are from one representative experiment.

Upon inoculation of LbCen-/- promastigotes in the ear dermis of BALB/c mice, parasites were detected after four days, at both the inoculation site and in dLNs (**Figures 8A** and **B**, respectively). However, LbCen-/- failed to induce lesion development, differently from mice inoculated with LbWT, in which lesions peaked at 4 weeks, then gradually subsided by 10 weeks (**Figures 8C**). Also, in mice infected with LbCen-/- promastigotes, parasites were not detected at the inoculation site, dLNs or in the spleen, 6 or 12 weeks post-inoculation (**Figures 8D** and **E**, respectively), indicating that LbCen-/- the impaired in vitro growth capacity (**Figure 7**) is recapitulated *in vivo*.

## Discussion

Since the first report of gene replacement in *Leishmania*, genetic manipulation has proven challenging due to its extensive genomic plasticity (Sterkers et al., 2014; Yagoubat et al., 2020), the presence of multicopy genes and polysomies (Rogers et al., 2011; Laffitte et al., 2016). The use of the functional *L. braziliensis* RNA interference (RNAi) machinery (Peacock et al., 2007; Lye et al., 2010) also showed off-target effects (Jackson et al., 2003), reinforcing that alternative approaches were necessary to achieve specific gene manipulation in this species. Herein, we used the LeishGEdit CRISPR toolbox (Beneke et al., 2017; Beneke and Gluenz, 2019) to generate a centrin-deficient *L. braziliensis*. Centrins play a fundamental role in centrosome duplication (Wiech et al., 1996) and centrin-deficient *leishmania* display abnormal cytokinesis, leading to the formation of multinucleated cells in axenic and intracellular amastigotes (Selvapandiyan et al., 2004). Centrin-deficient leishmania sp. are capable of inducing protection against leishmaniasis (Selvapandiyan et al., 2009; Bhattacharya et al., 2016; Zhang et al., 2020a) and are considered promising candidates for vaccine development.

The LeishGEdit CRISPR toolbox is a cloning-free, PCR-based CRISPR–Cas9 technology, involving the expression of sgRNAs *in vivo* based on T7-RNAP transcription (Beneke et al., 2017). This method allows for rapid and scalable manipulation of the *Leishmania* genome. The LeishGEdit CRISPR toolbox was first applied to *Leishmania* to generate immotile *L. mexicana*, deficient in PF16 (Beneke et al., 2017). Recently, Adaui et al. used this tool to generate a *L. braziliensis* cell line deficient in HSP-23 and HSP-100 (Adaui et al., 2020), recapitulating the phenotype observed in *L. donovani* HPS23 null mutants and showing the feasibility of CRISPR for the genetic manipulation of *L. braziliensis*. We build on these studies by using the LeishGEdit CRISPR toolbox for the generation of attenuated line of *L. braziliensis*, with the goal of developing an immunoprophylaxis tool against American Tegumentary Leishmaniasis. *L. braziliensis* is known for its genome plasticity (S L Figueiredo de Sá et al., 2019; Van Den Broeck et al., 2020). It is also the most biologically divergent Leishmania species (Peacock et al., 2007), causing a spectrum of clinical manifestations (Burza et al., 2018), that include mucosal and disseminated leishmaniasis, and accounting for the majority of CL cases in the Americas (David and Craft, 2009).

Centrins are calcium-binding cytoskeletal proteins involved in the centrosome duplication in eukaryotes (Salisbury, 1995; Salisbury et al., 2002). Centrin deletion using the LeishGEdit CRISPR toolbox was performed in a *L. braziliensis* clinical isolate (MHOM/BR/00/BA788) (De Moura et al., 2005) that does not harbor Leishmania RNA Virus (LRV1) (F. Novais, personal communication). After generating an *L. braziliensis* line expressing Cas9 and T7 RNAP, we performed deletion of both centrin alleles which were replaced by the neomycin and puromycin selectable markers. This was a different approach than that initially used to delete centrin in *L. donovani*, in which the mutant was generated by homologous recombination of centrin alleles (Selvapandiyan et al., 2001). Whole genome sequencing of *L. donovani* centrin*^−^*^/^*^−^* parasites revealed off-target deletions encompassing up to 6,900 bp in non-contiguous loci on several chromosomes, and also coding sequences (Gannavaram et al., 2017). Herein, whole genome sequencing of *LbCas9T7* and *LbCen^−/−^* confirmed the specific deletion of LbM22.1290 centrin locus in *LbCen^−/−^* mutant and no sign of translocation to other genomic location. Our analysis also does not suggest off-target effects.

Centrin-deficient *L. donovani* mutants do not replicate at the intracellular amastigote stage and parasites are selectively arrested, resulting in multinucleated parasites (Selvapandiyan et al., 2004). We corroborate these findings as *LbCen^−/−^* axenic amastigotes grow at a slower rate compared *LbWT* and, similar to *L. donovani*, present multi-nuclei (Selvapandiyan et al., 2004; Selvapandiyan et al., 2007). Further ultrastructural analyses confirmed that the morphological changes observed in *LbCen^−/−^* amastigotes were not present in WT counterparts or in *LbCas9T7*. As in centrin*^−^*^/^*^−^ L. donovani*, *LbCen^−/−^* presents pleomorphic and abnormally large cells, failed cytokinesis which, collectively, result in cell death (Selvapandiyan et al., 2004). With regards to survival in the host cell, a time course analysis showed that the parasite load of *LbCen^−/−^-*infected macrophages is significantly lower compared to cells infected with wild type *L. braziliensis*. The number of parasites internalized comparing *LbCen^−/−^* and *LbWT*, was similar indicating that centrin-defficiency does not alter entry capacity into the host cell (data not shown). Overall, we confirm that centrin deficiciency in *L. braziliensis* also impairs survival in the host cell.

Centrin*^−^*^/^*^−^ L. donovani* has been largely explored as an immunoprophylaxis tool. Upon *in vivo* infection, centrin*^−^*^/^*^−^ L. donovani* was rapidly and completely cleared from the spleen and liver of mice (Selvapandiyan et al., 2009). In hamsters, parasite burden was also lower in the spleen and undetectable in the liver, indicating safety of this attenuated line (Selvapandiyan et al., 2009). Centrin*^−^*^/^*^−^ L. braziliensis* recapitulates these findings as parasites are detected at an early time point (4 days) after inoculation into the ear dermis of mice but, later on, parasites are cleared. Thus, the impaired survival of *LbCen^−/−^* reported *in vitro* is also observed *in vivo*, confirming the attenuated infection profile. Indeed, BALB/c mice inoculated *LbCen^−/−^* failed to develop lesions throughout the course of experimentation, indicating that safety of this attenuated cell line, at least in an immunocompetent host.

Development of an effective prophylactic vaccine it of utmost importance to control leishmaniasis. Comparatively to *L. major*, however, *L. braziliensis* remains largely unexplored with regards to vaccine candidates, despite its importance as causative agent of mucosal and disseminated leishmaniasis. Although, centrin-deficient *L. donovani* protected against *L. braziliensis* in a mouse model (Selvapandiyan et al., 2009), whether this extends to the clinical spectrum of diseases caused by *L. braziliensis* remains to be determined. Moreover, vaccination with soluble *L. major* promastigote exogenous antigens did not protect against *L. braziliensis* infection (Tonui and Titus, 2007) nor did immunization with highly conserved leishmanial antigens (Salay et al., 2007). These results indicate that alternative strategies for inducing protection against *L. braziliensis* remain to be pursued. We build on existing literature by developing a centrin-deficient *L. braziliensis* cell line that shows attenuated behavior *in vitro* and *in vivo*. Future studies will address the capability of this cell line to confer protection against CL caused by *L. braziliensis*, namely, its ability to induce a poly-functional Th1 cellular response (Zhang et al., 2020a).

## Data Availability Statement

The datasets presented in this study can be found in online repositories. The names of the repository/repositories and accession number(s) can be found below: https://www.ncbi.nlm.nih.gov/, PRJNA763382.

## Ethics Statement

All animal experimentation was conducted in accordance with Guidelines for Animal Experimentation established by the Brazilian Council on Animal Experimentation (CONCEA). The local institutional review board (CEUA) approved all procedures involving animals (CEUA-015/2019-IGM/FIOCRUZ).

## Author Contributions

RS, FA-R, CF, JR-C, LM, PB, and SV performed experiments and analyzed data. RS, FA-R, DB, TB and CO drafted the manuscript. All authors listed have made a substantial, direct, and intellectual contribution to the work and approved it for publication.

## Funding

This work was supported by grants from the IGM-Fiocruz Bahia and the Fundação de Amparo a Pesquisa do Estado da Bahia (FAPESB). RS was supported by a travel grant from the Global Challenge Research Fund (GCRF), UK to participate at the 2nd Advanced School in Genetic Manipulation of Parasitic Protozoa, Federal University of Rio de Janeiro, Brazil. DB and CO are senior researchers at CNPq. LM received a fellowship from the CNPq. RS, FA-R, PB, and SV received a fellowship from the Coordenação de Aperfeiçoamento de Pessoal de Nível Superior—Brasil (CAPES)—Finance Code 001. TB was supported by an MRC PhD studentship (15/16_MSD_836338).

## Conflict of Interest

The authors declare that the research was conducted in the absence of any commercial or financial relationships that could be construed as a potential conflict of interest.

## Publisher’s Note

All claims expressed in this article are solely those of the authors and do not necessarily represent those of their affiliated organizations, or those of the publisher, the editors and the reviewers. Any product that may be evaluated in this article, or claim that may be made by its manufacturer, is not guaranteed or endorsed by the publisher.
