# Supplementary material for: Targeted Deletion of Centrin in Leishmania braziliensis Using CRISPR-Cas9-Based Editing
Source: Front Cell Infect Microbiol. 2022 Feb 17;11:790418. doi: 10.3389/fcimb.2021.790418 (PMC8892584; doi:10.3389/fcimb.2021.790418)
Supplement: Supplementary file 1 [file DataSheet_1.pdf]

Targeted deletion of Centrin in *Leishmania braziliensis* using CRISPR-Cas9-based editing

Rohit Sharma[1,¶], Francys Avendaño-Rangel[1,2,¶], Claudio Figueira[1], João Luís Reis-Cunha[3], Larissa Pinheiro Marques [4], Pedro B. Borba[1], Sayonara M. Viana[1,2], Tom Beneke[5], Daniella C. Bartholomeu[4], Camila I. de Oliveira[1,2,6*]

Supplemental Table 1. Primers used for centrin gene deletion and PCR based confirmation of KO in *Leishmania braziliensis*

Primers	Sequences
Upstream F	‘5-TTGCACTTATCGACAGCACTTTGCATACCAGTATAATGCAGACCTGCTGC-3’
Downstream R	‘5-CTTCTCGGGCTTGACCATCGCCGCCGCCCCCAATTTGAGAGACCTGTGC-3’
5’ sgRNA	GAAATTAATACGACTCACTATAGGGTCTATGACGCTGGCTTAAGGTTTTAGAGCTAGAAATAGC
3’sgRNA	GAAATTAATACGACTCACTATAGGCCTTCACCCTGCAGGAGTGAGTTTTAGAGCTAGAAATAGC
G00	AAAAGCACCGACTCGGTGCCACTTTTTCAAGTTGATAACGGACTAGCCTTATTTTAACTTGCTATTTCTAGCTCTAAAAC
ORF F Cen-/-	5' - GAG GCC TTC AAC CTC TTT GA - 3'
ORF R Cen-/-	5' - TCG GCG ATC ATC TCC TTT AG - 3'
NEO-F	5' - GTG CAA CCG TTC TAC AGT TC - 3'
NEO-R	5' - AAT AGC AGC CAG TCC CTT C - 3'
PAC-F	5' - TTC TCT CTT TTG TCC GTG ACT G - 3'
PAC-R	5' - TAC CAA TGT CCA AGC CCA CC - 3'

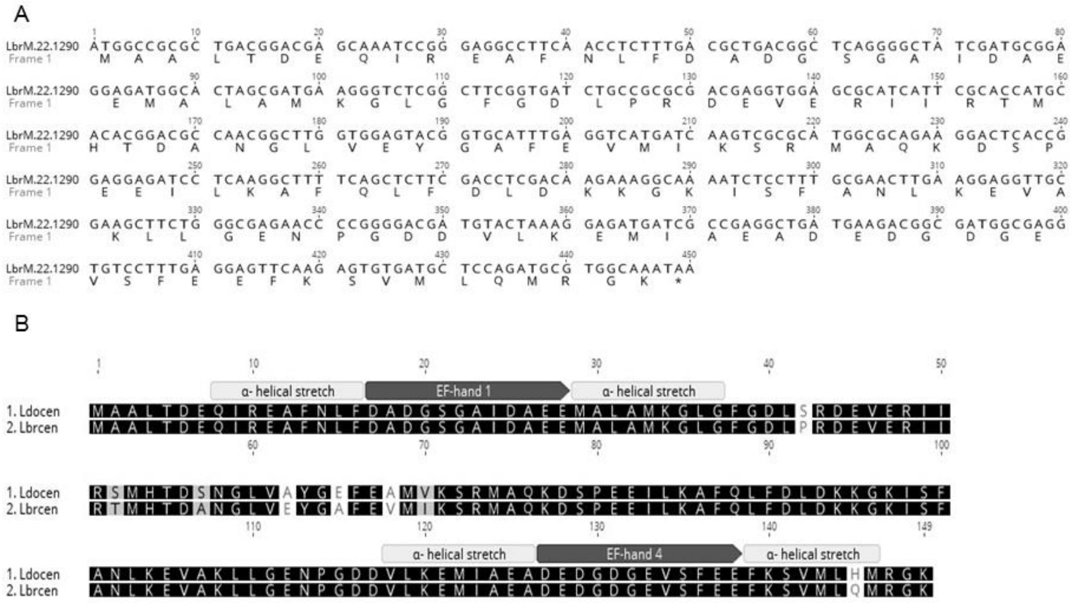

Supplemental Figure 1*. In silico* characterization of the *Leishmania braziliensis* centrin gene. (A) Nucleotide sequence (450 bp) and deduced amino acid sequence (149 aa) of the putative centrin gene in *L. braziliensis* LbrM22.190. (B) Amino acid sequence alignment *L. donovani* centrin (*Ldocen; Genbank accession number- AF406767)* and putative *L. braziliensis* centrin *(Lbrcen).* Boxes indicate high conservation at the calcium binding sites (EF-hand 1 and 4; alpha helical stretch).

A

> NEO.CENKO. F

30 nt Homology Flank (HF)

L. braziliesnis genomic sequence

NEO gene sequence

```
TKGGGSGTYYKRRRKGCTTGGTCGTCGTGTGTGATACACTCGCGCATGATGGAGCCGCCACGCAGAGATATTTCCC
TGTTGTACCTTGCACTTATCGACAGCACTTTGCATACCAGTATAATGCAGACCTGCTGCTGACCTACGCAGCCTTTT
GCGCACTTGGATCTCCTTTTGTCACGCTTGAGCAAAACGCTGCACCTCAAGCCGAGCATTCCCTTTCGTACGACAGG
AACAGCCGGGTGGTGGTGGTGGTAGGGGGAATGGTGATGATCAGATGCTTCCGAGATGCTGAACTCTCTCCCTCTC
GTACATCCTCTGCTTACCCTCTCCTTTTTTCTTCTCCATCGGCTGCATTCATGCTTTCTCCACAACGCCTTTGGCGTG
ACAAGAGCTCAGTCGCACTTGCGCTCCTCTTCTCGCCTCTGAAAAGGTCCTAGCTCTCTTCTGTTTTTTTTTTGTAAAA
GCAGCTCATACTCGAAGGCTATGGGATCGGCCATTGAACAAAATGGATTGCACGCAGGTTCTCCGGCCGCTTGGGT
GGAAAGACTATTCGGCTATGACTGGGCACAACAAACAATCGGCTGCTCTGATGCCGCCGTGTTCCGGCTGTCAGCG
CAGGGGCGCCCGGTTCTTTTTGTCAAGACCGACCTGTCCGGTGCCCTGAATGAACTGCAGGACGAGGCAGCGCGG
CTATCGTGGCTGGCCACAACGGGCGTTCCTTGCGCAGCTGTGCT
```

B

>PURO.PAC.F

30 nt Homology Flank (HF)

L. braziliesnis genomic sequence

PAC gene sequence

```
TSYSGSSSGYYCKGMMTGSCTTCGTCGTCGTGTGTGATACACTCGCGCATGATGGAGCCGCCACGCAGAGATATTTCCC
TGTTGTACCTTGCACTTATCGACAGCACTTTGCATACCAGTATAATGCAGACCTGCTGCAGCCGTTGCATTGGTGGTCGT
CTCTGCCGCTGCCCCCCCCCCCTTCCCCGGCTAAACCKGGGKACCTTCGGGGGGGGGGTATCCCCGGCTTCCCAACC
CTTGGTTGGGGGAAAAGGGTTGGGGGCCTTCCACCGTCAAAAACCGGGGGAAAAGGGGCACGCCCGCCCCCCCAAGG
GGCCCGAAATATTACGAGGGGAATCCCATCCCCACGAAGGGGCCAAAAAACTAATTTTTTTTTTTGGCGAACCCCCCCCC
CCTTTTTTTTTGGTTTTAAATGGGCCCCCCCCCCCCTACCGGTTTTTCTCCAAAATTTAAACAAAAAACCAATTTACCCCCC
CAACTAACTCCCAATCCTCCCCGGGGCGAACAATCCATTTCGCCTTCCCCCTTTGAACAAAAACCCCCCCATTCCCCCAT
TTTTTTTTGCGGTCCTCCATAATGACYGGATACAACCAACCGGTCCTCTTGCTACCCGGAYATGGGCCCGGGCGWCCGA
CCTGGTGGTTGCTTCCTGATATCCGGCACCGACTACCGTGAACAATAGGACATTGACCAGTCAGAACTTCCAAGTATCTA
ACCGTGGCTGACMTTGTTA
```

Supplemental Figure 2. Confirmation of the correct integration of antibiotic donor DNA within the Centrin locus. (A) and (B) annotation of the sequences obtained from the Neo and PAC sequenced amplicons (respectively), derived from the diagnostic PCR.
